# Supplementary material for: Impact of COVID-19 epidemic curtailment strategies in selected Indian states: An analysis by reproduction number and doubling time with incidence modelling
Source: PLoS One. 2020 Sep 16;15(9):e0239026. doi: 10.1371/journal.pone.0239026 (PMC7494123; doi:10.1371/journal.pone.0239026)

Supplementary Appendix 1: R Code

Contents

Introduction **2**

Loading the relevant packages **2**

Load Data **2**

Description of the dataset **3**

Manipulation of date formats and dichotomizing variables **4**

Selecting of States **4**

Creating incidence objects by state by different phases of epidemic **5**
- Before Lockdown (2020-03-25) 5
- 15 days into lockdown (2020-04-08) 5
- 30 days into lockdown (2020-04-08) 5

Serial Interval **6**

Estimating Transmission Parameters **6**
- 14 days Post-Lockdown 6
- 30 days Post-Lockdown 7

Plotting change in transmission parameters by state **9**
- Doubling Time 9
- Growth Rate 10
- Reproduction Number 10

Plotting Daily and Cumulative Incidence **11**

Introduction

This document contains all the R code used to perform the analysis including data wrangling and estimation of transmission parameters. The code can be executed within the R Notebook which displays the results in-line beneath the code.

Loading the relevant packages

```
library(ggplot2)
library(RJSONIO)
library(dplyr)
library(incidence)
library(earlyR)
library(R0)
library(projections)
library(distcrete)
library(knitr)
opts_chunk$set(tidy.opts=list(width.cutoff=30),tidy=TRUE)
```

Load Data

The raw data is downloaded from the crowd-sourced website COVID-19 India Tracker. The two files are `raw_data1.json` containing data till 19th April 2020 and `raw_data2.json` containing data till 26th April 2020. The following code converts them into class `data.frame` and merges them into one.

```
json_file = "C:/Users/Arun/Dropbox/COVID-19/SEIR/raw_data1.json"
#json_file = "https://api.covid19india.org/raw_data1.json"
json_file2 = RJSONIO::fromJSON(json_file)
json_file2 = json_file2[["raw_data"]]
json_file3 <- lapply(json_file2, function(x) {
  x[sapply(x, is.null)] <- NA
  unlist(x)
})

df <- do.call("rbind", json_file3)

names <- colnames(df)
df <- as.data.frame(df)
names(df) <- names
keep <- c("dateannounced",
          "detectedstate",
          "statecode",
          "detectedcity",
          "currentstatus",
          "statuschangedate",
          "typeoftransmission")
df1<- df[keep]

json_file = "C:/Users/Arun/Dropbox/COVID-19/SEIR/raw_data2.json"
#json_file = "https://api.covid19india.org/raw_data2.json"
```

```r
json_file2 = RJSONIO::fromJSON(json_file)
json_file2 = json_file2[["raw_data"]]
json_file3 <- lapply(json_file2, function(x) {
  x[sapply(x, is.null)] <- NA
  unlist(x)
})

df <- do.call("rbind", json_file3)

names <- colnames(df)
df <- as.data.frame(df)
names(df) <- names
keep <- c("dateannounced",
          "detectedstate",
          "statecode",
          "detectedcity",
          "currentstatus",
          "statuschangedate",
          "typeoftransmission")
df2<- df[keep]

json_file = "C:/Users/Arun/Dropbox/COVID-19/SEIR/raw_data3.json"
#json_file = "https://api.covid19india.org/raw_data3.json"
json_file2 = RJSONIO::fromJSON(json_file)
json_file2 = json_file2[["raw_data"]]
json_file3 <- lapply(json_file2, function(x) {
  x[sapply(x, is.null)] <- NA
  unlist(x)
})

df <- do.call("rbind", json_file3)

names <- colnames(df)
df <- as.data.frame(df)
names(df) <- names
keep <- c("dateannounced",
          "detectedstate",
          "statecode",
          "detectedcity",
          "currentstatus",
          "statuschangedate",
          "typeoftransmission")
df3 <- df[keep]

df_backup <- rbind.data.frame(df1, df2, df3)
df <- df_backup
```

Description of the dataset

The dataset has 32948 rows and 7 columns. The variables selected are "dateannounced", "detectedstate", "statecode", "detectedcity", "currentstatus", "statuschangedate", and "typeoftransmission". The "dateannounced" variable has the date of announcement of the case. The "detectedstate" and "detectedcity" variables

have the name of the state and city in which the case was detected and "statecode" represents the initials of the state. The "currentstatus" column represents the status of the patient i.e. Hospitalized, Recovered, Deceased or Migrated.

Manipulation of date formats and dichotomizing variables

The `dateannounced` variable is then coherced `as.Date()` into the format `%d/%m/%Y`. The same is done for the variable `statuschangedate`. The variable `typeoftransmission` represents the nature of origin of the case i.e. 'Local' or 'Imported'. All cases occuring after 9 March 2020 are considered as local cases.

```
df$dateannounced <- as.Date(df$dateannounced, "%d/%m/%Y")
df$statuschangedate<- as.Date(df$statuschangedate, "%d/%m/%Y")
df$typeoftransmission <- ifelse(df$typeoftransmission == "Imported"
                                | df$typeoftransmission ==  "Imported ",
                                "Imported", "Local")
df$typeoftransmission <- ifelse(df$typeoftransmission == "Imported"
                                & df$dateannounced > as.Date("2020-04-09"),
                                "Local", df$typeoftransmission)
```

Selecting of States

The top 10 states with highest number of cases as of 23rd April 2020 were selected and saved as an object `states_keep`. This object is used to pass through the `filter()` function of the `dplyr` package to select the states mentioned in the object. The function `droplevels.data.frame()` was used to drop the levels from the subsetted dataset.

```
d1 <- df %>%
  filter(typeoftransmission == "Local") %>%
  filter(dateannounced <= "2020-04-23")%>%
  group_by(detectedstate) %>%
  summarize(n = n()) %>%
  arrange(-n)
#View(d1)

d2 <- df %>%
  filter(dateannounced <= "2020-04-23")%>%
  group_by(detectedstate) %>%
  summarize(n = n()) %>%
  arrange(-n)
#View(d2)

states_keep <- factor(d2$detectedstate[1:10],
                      levels = d2$detectedstate[1:10])

# List of States Selected
states_keep
```

```
##  [1] Maharashtra    Gujarat        Delhi          Rajasthan      Madhya Pradesh
##  [6] Tamil Nadu     Uttar Pradesh  Telangana      Andhra Pradesh West Bengal
## 10 Levels: Maharashtra Gujarat Delhi Rajasthan Madhya Pradesh ... West Bengal
```

```
# Subset the dataframe by keeping only the data selected states
df <- df %>%
  filter(detectedstate%in% states_keep) %>%
  droplevels.data.frame()
```

Creating incidence objects by state by different phases of epidemic

The incidence objects were created baed on the three phases of the epidemic. The function `incidence()` from the R package `incidence` was used for this purpose. The phases of the epidemic that were considered for the creation of the incidence objects were:

1. Before Lockdown (2020-03-25)
2. Early Epidemic i.e. 15 days into lockdown (2020-04-08)
3. Current timeframe i.e. 30 days into lockdown (2020-04-23)

This is acheived by executing a `for` loop. This produces three lists containing 10 incidence objects each (one for each timeframe and one for each of the 10 selected states).

Before Lockdown (2020-03-25)

```
incid_before_LD <- NULL
data <- NULL
for(i in unique(states_keep)){
  data[[i]] <- subset(df, detectedstate == i)

  ## Create an incidence object
  incid_before_LD[[i]] <- incidence(data[[i]]$dateannounced)
  incid_before_LD[[i]] <- subset(incid_before_LD[[i]], to = "2020-03-25")
}
```

15 days into lockdown (2020-04-08)

```
incid_15_LD <- NULL
data <- NULL
for(i in unique(states_keep)){
  data[[i]] <- subset(df, detectedstate == i)

  ## Create an incidence object
  incid_15_LD[[i]] <- incidence(data[[i]]$dateannounced)
  incid_15_LD[[i]] <- subset(incid_15_LD[[i]], to = "2020-04-08")
}
```

30 days into lockdown (2020-04-08)

```
incid_30_LD <- NULL
data <- NULL
for(i in unique(states_keep)){
  data[[i]] <- subset(df, detectedstate == i)
```

```
  ## Create an incidence object
  incid_30_LD[[i]] <- incidence(data[[i]]$dateannounced)
  incid_30_LD[[i]] <- subset(incid_30_LD[[i]], to = "2020-04-23")
}
```

Serial Interval

Since the generation time for COVID-19 cases was difficult to obtain, we used the Serial Interval for COVID-19 as a proxy for the generation time.The serial interval is created using the `generation.time()` function in the `R0` package where the distribution assumes a gamma distribution with a mean of 4.4 days and a standard deviation of 3 days. This is saved as a discretized GT object `mGT`. This object is then passed to other functions to estimate the reproduction number.

```
mGT <- generation.time("gamma", c(4.4, 3))
```

Estimating Transmission Parameters

The following code is passed for all the ten incidence objects in three lists using a `for` loop. The following code estimates the transmission parameters including growth rate, doubling time and time-varyintg reproduction number $R_{(t)}$.

14 days Post-Lockdown

```
# 14 days Post LockDown Epidemic Phase
# Date= 2020-04-08"
incid_14 <- NULL
for(i in unique(states_keep)){
  data[[i]] <- subset(df, detectedstate == i)
  ## Create an incidence object
  incid_14[[i]] <- incidence(data[[i]]$dateannounced)
  incid_14[[i]] <- subset(incid_14[[i]], to = "2020-04-08")
}

incid_state <- NULL
f_state <- NULL
dt_state <- NULL
gr_state <- NULL
cases_state <- NULL
res_state <- NULL
r0_state <- NULL
tab <-  NULL

for(i in 1:10){
  incid_state[[i]] <- incid_14[[i]]
  f_state[[i]] <- fit(incid_state[[i]])
  dt_state[[i]] <- c(f_state[[i]][[2]][4], as.numeric(f_state[[i]][[2]][5][[1]]))
  gr_state[[i]] <- c(f_state[[i]][[2]][1], as.numeric(f_state[[i]][[2]][2][[1]]))
  cases_state[[i]] <- as.numeric(incid_state[[i]]$counts[,1])
  mGT <- generation.time("gamma", c(4.4, 3))
```

```
  res_state[[i]] <- est.R0.ML(cases_state[[i]], mGT)
  r0_state[[i]] <- c(res_state[[i]]$R, res_state[[i]]$conf.int)
  tab[[i]] <- rbind(r0_state[[i]],dt_state[[i]],gr_state[[i]])
}
```

```
## Warning in fit(incid_state[[i]]): 9 dates with incidence of 0 ignored for
## fitting

## Warning in fit(incid_state[[i]]): 12 dates with incidence of 0 ignored for
## fitting

## Warning in fit(incid_state[[i]]): 3 dates with incidence of 0 ignored for
## fitting

## Warning in fit(incid_state[[i]]): 11 dates with incidence of 0 ignored for
## fitting

## Warning in fit(incid_state[[i]]): 8 dates with incidence of 0 ignored for
## fitting

## Warning in fit(incid_state[[i]]): 11 dates with incidence of 0 ignored for
## fitting

## Warning in fit(incid_state[[i]]): 7 dates with incidence of 0 ignored for
## fitting

## Warning in fit(incid_state[[i]]): 8 dates with incidence of 0 ignored for
## fitting
```

```
tab <- do.call("rbind.data.frame",tab)
tab$time <- rep("14 days", 30)
tab$parameter <- rep(c("r0", "dt", "gr"),10)
tab$state <- rep(states_keep, each = 3)
tab <- tab[c(6,4,5,1,2,3)]
names(tab) <- c("state", "time", "parameter", "estimate", "lci", "uci")
tab$estimate <- unlist(tab$estimate)
tab$lci <- unlist(tab$lci)
tab$uci <- unlist(tab$uci)
transmission_params_14 <- tab
```

30 days Post-Lockdown

```
# 30 days Post LockDown Epidemic Phase
# Date= 2020-04-23"
incid_30 <- NULL
for(i in unique(states_keep)){
  data[[i]] <- subset(df, detectedstate == i)
  ## Create an incidence object
  incid_30[[i]] <- incidence(data[[i]]$dateannounced)
  incid_30[[i]] <- subset(incid_30[[i]], to = "2020-04-23")
}

incid_state <- NULL
f_state <- NULL
dt_state <- NULL
gr_state <- NULL
```

```r
cases_state <- NULL
res_state <- NULL
r0_state <- NULL
tab <-  NULL

for(i in 1:10){
  incid_state[[i]] <- incid_30[[i]]
  f_state[[i]] <- fit(incid_state[[i]])
  dt_state[[i]] <- c(f_state[[i]][[2]][4], as.numeric(f_state[[i]][[2]][5][[1]]))
  gr_state[[i]] <- c(f_state[[i]][[2]][1], as.numeric(f_state[[i]][[2]][2][[1]]))
  cases_state[[i]] <- as.numeric(incid_state[[i]]$counts[,1])
  mGT <- generation.time("gamma", c(4.4, 3))
  res_state[[i]] <- est.R0.ML(cases_state[[i]], mGT)
  #res_state[[i]] <- est.R0.SB(cases_state[[i]], mGT)
  r0_state[[i]] <- c(res_state[[i]]$R, res_state[[i]]$conf.int)
  tab[[i]] <- rbind(r0_state[[i]],dt_state[[i]],gr_state[[i]])
  #names(tab[[i]]) <- c("estimate", "lci" ,"uci")
}
```

```
## Warning in fit(incid_state[[i]]): 9 dates with incidence of 0 ignored for
## fitting

## Warning in fit(incid_state[[i]]): 12 dates with incidence of 0 ignored for
## fitting

## Warning in fit(incid_state[[i]]): 3 dates with incidence of 0 ignored for
## fitting

## Warning in fit(incid_state[[i]]): 11 dates with incidence of 0 ignored for
## fitting

## Warning in fit(incid_state[[i]]): 8 dates with incidence of 0 ignored for
## fitting

## Warning in fit(incid_state[[i]]): 11 dates with incidence of 0 ignored for
## fitting

## Warning in fit(incid_state[[i]]): 7 dates with incidence of 0 ignored for
## fitting

## Warning in fit(incid_state[[i]]): 8 dates with incidence of 0 ignored for
## fitting
```

```r
tab <- do.call("rbind.data.frame",tab)
tab$time <- rep("30 days", 30)
tab$parameter <- rep(c("r0", "dt", "gr"),10)
tab$state <- rep(states_keep, each = 3)
tab <- tab[c(6,4,5,1,2,3)]
names(tab) <- c("state", "time", "parameter", "estimate", "lci", "uci")
tab$estimate <- unlist(tab$estimate)
tab$lci <- unlist(tab$lci)
tab$uci <- unlist(tab$uci)
transmission_params_30 <- tab
```

Plotting change in transmission parameters by state

The summary tables created in the previous code chunk are recalled to plot the change in transmission parameters by state. For the purpose of these plots we will only use the two timeframes after lockdown. The reason for omiting the timeframe before lockdown is due to the wide confidence intervals and inadequate number of cases during this period to calculate robust estimates.

```
# Aggregate the timepoints after enforcment of lockdown
plot_states_14 <- transmission_params_14
plot_states_30 <- transmission_params_30

plot_params <- rbind.data.frame(plot_states_14, plot_states_30)
plot_params$time <- c(rep("15 days",30),
                      rep("30 days",30))

plot_params$time <- factor(plot_params$time,
                           levels = c("15 days",
                                      "30 days"))
```

Doubling Time

```
plot_params_dt <- plot_params %>%
  filter(parameter == "dt")

ggplot(plot_params_dt, aes(x = factor(time, levels = levels(plot_params$time)),
                           y = estimate,
                           group = state)) +
  geom_point(aes(colour = state)) +
  geom_line(aes(colour = state)) +
  facet_wrap(~state, nrow = 2, scales = "free_y") +
  scale_y_continuous(name = "Doubling Time", limits = c(3,11)) +
  scale_x_discrete(name = "Time Since Lock Down") +
  theme(legend.position = "none",
        axis.text.x = element_text(size=8) )
```

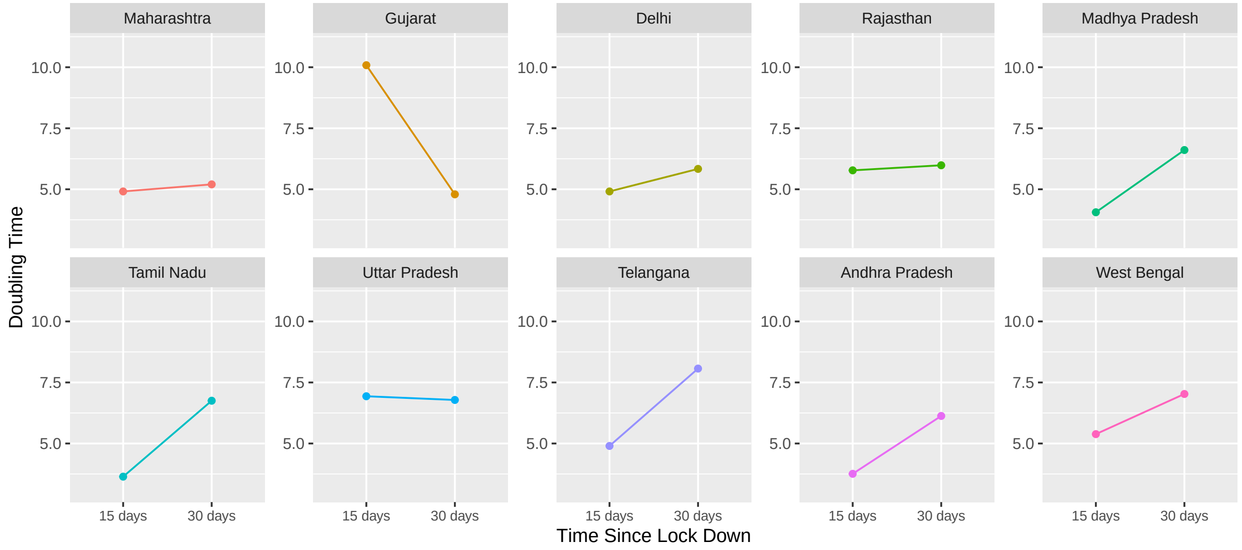

Growth Rate

```r
plot_params_gr <- plot_params %>%
  filter(parameter == "gr")

ggplot(plot_params_gr, aes(x = factor(time, levels = levels(plot_params$time)),
                           y = estimate, group = state)) +
  geom_point(aes(colour = state)) +
  geom_line(aes(colour = state)) +
  facet_wrap(~state, nrow = 2) +
  scale_y_continuous(name = "Growth Rate") +
  scale_x_discrete(name = "Time Since Lock Down") +
  theme(legend.position = "none",
        axis.text.x = element_text(size=8) )
```

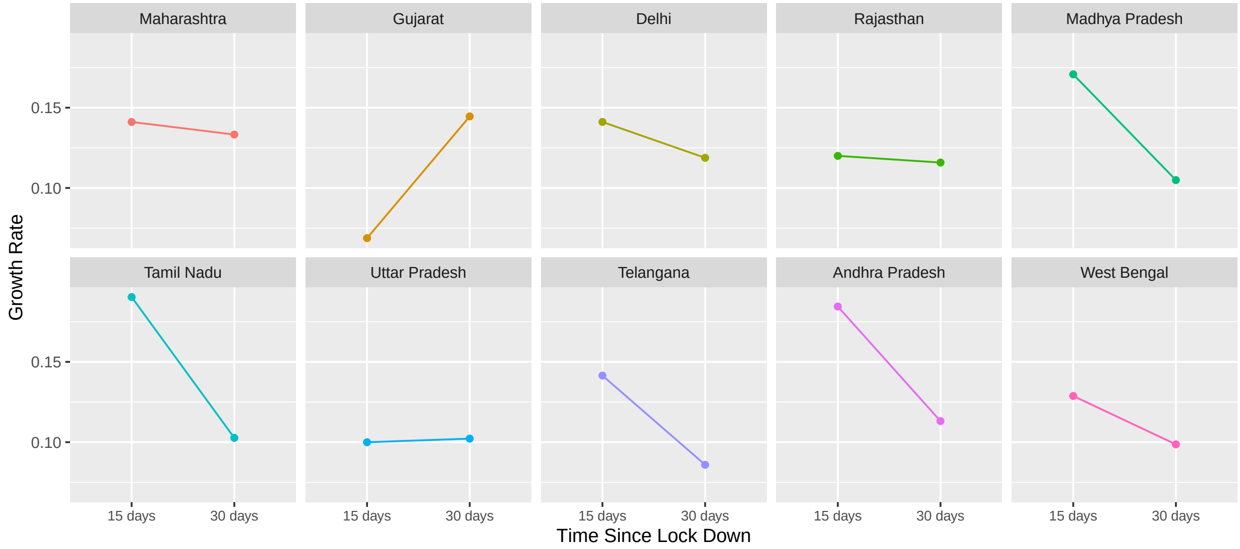

Reproduction Number

```r
plot_params_r0 <- plot_params %>%
  filter(parameter == "r0")

ggplot(plot_params_r0, aes(x = factor(time, levels = levels(plot_params$time)),
                           y = estimate, group = state)) +
  geom_point(aes(colour = state)) +
  geom_line(aes(colour = state)) +
  geom_hline(yintercept = 1, color = "red", linetype = "dashed", alpha = 0.2) +
  facet_wrap(~state, nrow = 2) +
  scale_y_continuous(name = "Reproduction Number", limits = c(0,6)) +
  scale_x_discrete(name = "Time Since Lock Down") +
  theme(legend.position = "none",
        axis.text.x = element_text(size=8) )
```

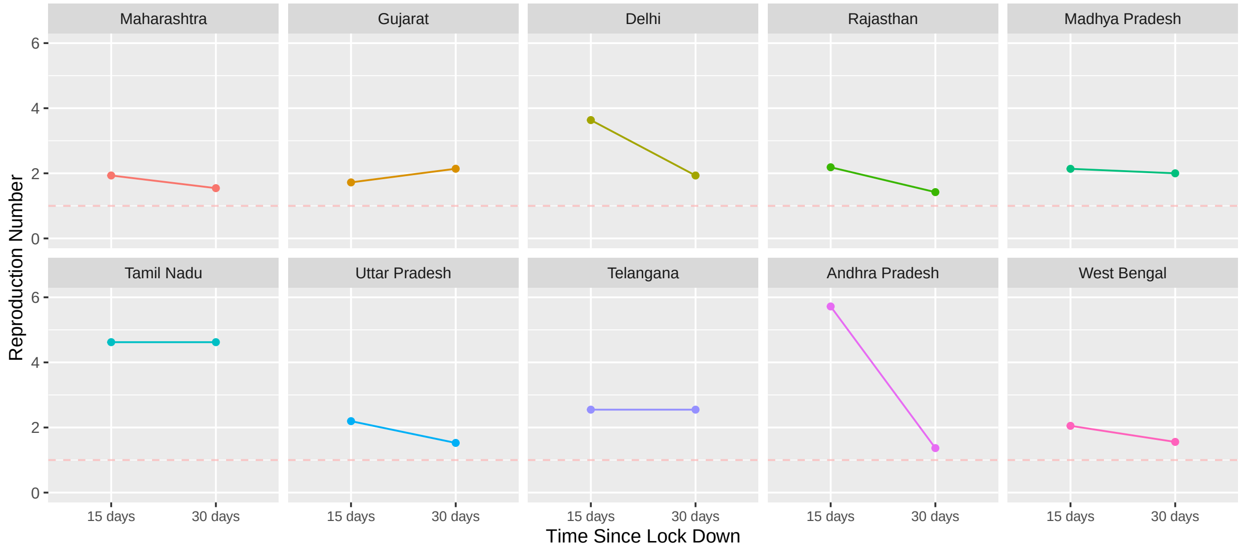

Plotting Daily and Cumulative Incidence

The following code produces the composite plot reported in the manuscript. It has dates on the x-axis and daily incidence on the primary y-axis and cumulative incidence on the secondary y-axis. The plot is divided into three regions based on the timeframes. Each state is represented by a colour and their trend in cumulative cases can be seen.

```
library(reshape2)
library(ggplot2)
library(RColorBrewer)
library(wesanderson)

incid_data <- incidence(df$dateannounced, groups = df$detectedstate)
incid_data <- subset(incid_data, to = "2020-04-25")

incid_data1 <- data.frame(incid_data)
incid_data2 <- melt(incid_data1  , id.vars=c("dates"))


cumulate_data <- cumulate(incid_data)
cumulate_data1 <- data.frame(cumulate_data)
cumulate_data2 <- melt(cumulate_data1 , id.vars=c("dates"))

composite_plot <- cbind.data.frame(incid_data2, cumulative = cumulate_data2$value)
names(composite_plot) <- c("Dates", "States", "Incidence", "Cumulative")

col1 <- brewer.pal(10, "Paired")
col2 <- wes_palette("Zissou1", 10, type = "continuous")

p <- ggplot(composite_plot, aes(x = Dates, y = Incidence)) +
  geom_bar(aes(fill = States), alpha = 0.5, stat = 'identity') +
  geom_line(mapping = aes(x = Dates, y = Cumulative/3, colour = States), size = 1) +
  geom_vline(xintercept = as.numeric(composite_plot$Dates[c(23,39,53)]),
             colour=c("red", "black","black"), linetype=4) +
```

```
  geom_text(aes(x=composite_plot$Dates[12], label="Before Lockdown", y=2500),
            colour="blue", size=3) +
  geom_text(aes(x=composite_plot$Dates[22], label="Lockdown (25th March 2020)", y=600),
            angle = 90, size=3) +
  geom_text(aes(x=composite_plot$Dates[31], label="15 Days \n since Lockdown", y=2500),
            colour="blue", size=3) +
  geom_text(aes(x=composite_plot$Dates[46], label="30 Days \n since Lockdown", y=2500),
            colour="blue", size=3) +
  scale_y_continuous(name = "Daily Incidence",
                     sec.axis = sec_axis(~.*3, name = "Cumulative Incidence")) +
  scale_colour_manual(values = col1) +
  scale_fill_manual(values = col1)+
  theme_set(theme_gray() + theme(legend.key=element_blank(),legend.position = "bottom"))
p
```

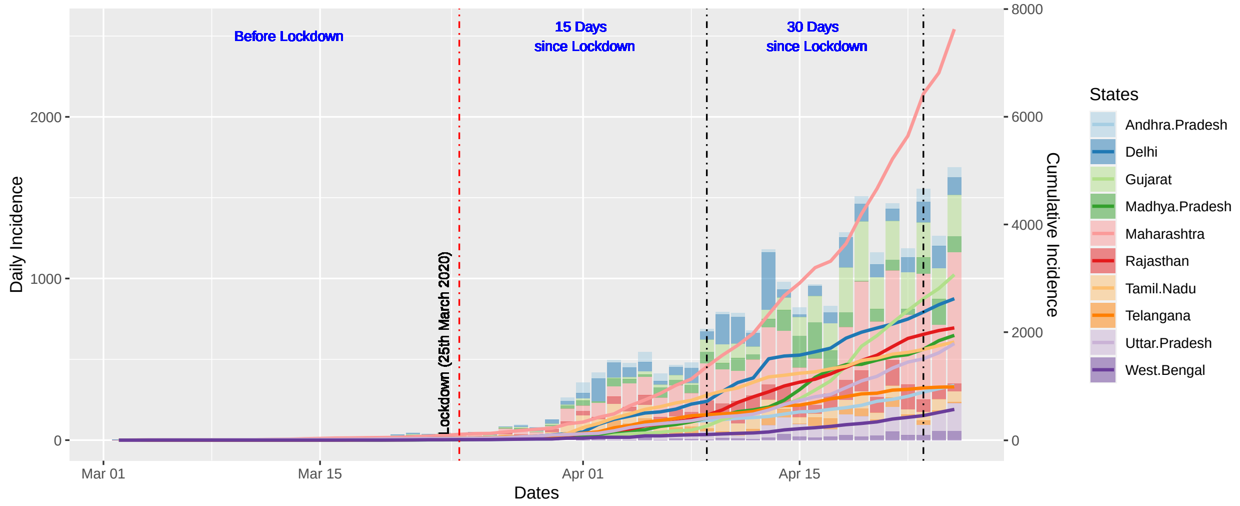

Supplement: S1 Appendix — (PDF) [file pone.0239026.s001.pdf]
