# Supplementary material for: Impact of COVID-19 epidemic curtailment strategies in selected Indian states: An analysis by reproduction number and doubling time with incidence modelling
Source: PLoS One. 2020 Sep 16;15(9):e0239026. doi: 10.1371/journal.pone.0239026 (PMC7494123; doi:10.1371/journal.pone.0239026)
Supplement: S1 Table — (PDF) [file pone.0239026.s003.pdf]

Supplementary Table 1

The table below shows the number of imported and local cases by time period and the number of days between first case and lockdown. About half of the states reported less than 10 local cases before lockdown. The estimates of transmission parameters before lockdown including R0, doubling time and growth rate may be heavily influenced by the high proportion of imported cases.

We considered the baseline time period to be 15 days of lockdown as the influence of imported cases on estimation of R0 after lockdown would be diminished due to travel restrictions both inter and intra state. This was the rationale behind omitting the time period of before lockdown in estimation of transmission parameters.

State	Date of first case reporting (Days between first case and lockdown)		No. of new cases in different time periods					
			Before Lockdown		*15 days Lockdown*		*30 days Lockdown*	
	Imported	*Local*	*Imported*	*Local*	*Imported*	*Local*	*Imported*	*Local*
Maharashtra	9th March (16 days)	10th March (15 days)	53	54	8	903	0	4631
Gujarat	19th March (6 days)	21st March (4 days)	20	16	2	137	0	2232
Delhi	2nd March (23 days)	9th March (16 days)	14	16	2	544	0	1672
Rajasthan	3rd March (22 days)	20th March (5 days)	11	21	33	278	0	1545
Madhya Pradesh	20th March (5 days)	22nd March (3 days)	5	2	3	280	0	1297
Tamil Nadu	7th March (18 days)	18th March (7 days)	15	3	13	659	0	939
Uttar Pradesh	5th March (20 days)	4th March (21 days)	17	18	1	296	0	1117
Telangana	2nd March (23 days)	21st March (4 days)	34	5	1	364	0	539
Andhra Pradesh	12th March (13 days)	22nd March (3 days)	7	1	3	303	0	499
West Bengal	17th March (8 days)	21st March (4 days)	5	4	1	81	0	332
