# Supplementary material for: Impact of COVID-19 epidemic curtailment strategies in selected Indian states: An analysis by reproduction number and doubling time with incidence modelling
Source: PLoS One. 2020 Sep 16;15(9):e0239026. doi: 10.1371/journal.pone.0239026 (PMC7494123; doi:10.1371/journal.pone.0239026)
Supplement: S2 Table — (PDF) [file pone.0239026.s004.pdf]

Supplementary Table 2

The table below shows the number of cases predicted vs number of cases observed.

State	Cumulative Incidence as on 23rd April 2020	Predicted (10th day Cumulative Incidence i.e. On 3rd May 2020)	Observed (3rd May 2020)	Percent Increase in last 10 days
**Maharashtra**	6427	10362 - 16825	12974	102%
**Gujarat**	2624	4186 - 6798	5428	107%
**Delhi**	2376	4355 - 7073	4549	91%
**Rajasthan**	1964	3434 - 5583	2886	47%
**Madhya Pradesh**	1687	3070 - 5001	2837	68%
**Tamil Nadu**	1683	3186 - 5179	3023	80%
**Uttar Pradesh**	1510	2596 - 4214	2645	75%
**Telangana**	970	1831 - 2977	1082	12%
**Andhra Pradesh**	893	1538 - 2496	1583	77%
**West Bengal**	456	764 - 1240	1198	163%
