# Supplementary material for: Impact of COVID-19 epidemic curtailment strategies in selected Indian states: An analysis by reproduction number and doubling time with incidence modelling
Source: PLoS One. 2020 Sep 16;15(9):e0239026. doi: 10.1371/journal.pone.0239026 (PMC7494123; doi:10.1371/journal.pone.0239026)
Supplement: Attachment — Submitted filename: Response to Reviewers.docx [file pone.0239026.s006.docx]

Response to Reviewers

At the outset we would like to thank the editor and the reviewers for their valuable comments, it is indeed an honour to receive critique and suggestions from esteemed scholars like you. We are also grateful for giving us the motivation continue playing our little part in fighting the COVID-19 epidemic.

# Editor Comments:

1. Please ensure that your manuscript meets PLOS ONE's style requirements, including those for file naming. The PLOS ONE style templates can be found at https://journals.plos.org/plosone/s/file?id=wjVg/PLOSOne_formatting_sample_main_body.pdf and https://journals.plos.org/plosone/s/file?id=ba62/PLOSOne_formatting_sample_title_authors_affiliations.pdf

**Response:**

**The authors apologize for the errors in the submission of the manuscript. We have formatted and resubmitted the manuscript according to the PLOS ONE style template as suggested.**

### Additional Editor Comments:

Basically I agree with the points both reviewers pointed out then they should be addressed before publication.

**Response:**

**We attempted to address all the issues raised by the editor and reviewers to the best of our abilities. We have performed a thorough literature review and rewritten the methods and discussion part, incorporating all the suggestions. We also described the R code and the accompanying files in greater detail, updated the Supplementary Appendix. We published the steps employed in the computational workflow of the analysis at protocols.io and uploaded data and other relevant files adhering to the PLOS One’s publication policy.**

# Reviewer comments:

# Reviewer #1:

There are a number of small issues in the writing which should be cleared up before publication, but overall the paper is readable and reasonably constructed.

**Response:**

**We thank the Reviewer for their encouraging words. We have taken all your suggestions and rectified the manuscript. We hope we addressed the issues pointed adequately.**

## Point 1:

The bigger issue is that relatively little information is given about the actual methodology, the benefits and drawbacks, alternatives, and general conclusions. Two R packages are used to fit epidemic models to a particular data source, but the methods employed in each are not really described in any detail. Some statements are extremely vague: "This method optimizes β and S0 from the sequence of binomial likelihood with the fundamental assumptions of conditional independence" - that's not enough information to describe the form of the likelihood, or the maximization scheme. The language describing the methods remains confusing into the discussion section ("yet the methods are robust in terms of conditional independence and MCMC methods used to tackle the Bayesian influence").

**Response:**

**The authors thank the Reviewer for his/her suggestions. We have rewritten the Methodology section in a more lucid language and also tried to explain the rationale behind our methodology. We also elaborated on the considerations and assumptions in greater detail and included the detailed descriptions of the elements of the R code in the Supplementary Appendix.**

**The relevant part of the Methdology Section is given below:**

**Estimation of Reproduction Number**

The time-varying reproductive number (R(t)) was estimated by using the maximum likelihood (ML) method. [13] This method presumes that all the secondary cases linked to the primary cases follow a Poisson process (event rate is constant) and the corresponding serial interval follows a multinomial distribution. This leads to a gradual trend towards zero secondary cases (with multitude of time steps) arising from primary cases during a specific time-step. The gradient of depends on the parameter distribution function (PDF) of the serial interval. The “est R0.ML()” function in the “*R0*” package. This runs an expectation maximum (EM) algorithm which maximises the distribution probability of primary and secondary cases in reference to time. This method assumes that infectee always develops symptoms only after infector thus the value of the serial interval will be positive.

**Serial Interval**

For the parameter distribution function (PDF), we could not obtain the generation time (time lag between the infection in primary case and secondary cases) distribution directly with infector-infectee pairs due to the lack of data availability. Therefore, it was substituted with the serial interval distribution discretized on a 1-day time step. This was created using the generation.time()” function in the “R0” package. For parametrisation purposes we chose a gamma distribution as it accommodates for the underlying changing number of events in the constant event rate (Poisson process). The distribution assumptions were aligned with the emerging literature as well as the observed plausible transmission dynamics. The mean and standard deviation for serial interval approximations were 4.4 days and 3 days respectively.[14] The shape (number of events in time step) and scale (the reciprocal of event rate) of the distribution were 2.15 and 2.04 respectively.

## Point 2:

The data itself is described as "crowd sourced", but this is not sufficiently descriptive. The data appears to be collected from official sources by volunteers - that's fine, but a discussion of the limitations of official figures is certainly important with COVID-19. Testing and ascertainment has changed throughout the course of the pandemic, but the potentially incomplete data are simply treated as known measures of incidence here.

**Response:**

**The authors agree with the Reviewer’s comment regarding the insufficient description of the data source.**

**We have added the following text in the Methdology section and hope it describes the data source sufficiently and addresses the concerns raised:**

**Data Source**

The data source is a crowd-sourced database maintained at www.covid19india.org. [6] The website is maintained by a group of volunteers who curate and verify the data coming from several sources. The data is compiled from the state bulletins, official handles of state governments, and health ministries. It is validated, updated periodically and published into an application programming interface (API) and Google spreadsheet which is accessible at api.covid19india.org for the public. Apart from the patient-level data, the API includes district-level, state-level, and national-level datasets. We used the data from the line-listing of the cases reported as positive for COVID-19. The data was iteratively and progressively accessed through the database in coherence with creation and improvement in analysis code. The last access to database was made on 1^st^ May, 2020. We truncated the data up to 23^th^ April 2020 for the purpose of this study. This buffer period of 7 days offered some immunity against the possible delay to add the cases and our limitation to access the data in real time.

**We also attempted to address the implications of testing, quality of the data on the estimation of these vital parameters in the Discussion section. The relevant portion of the manuscript is given below:**

The credibility of a crowd-sourced dataset may be viewed from the following probes: under-reporting, duplicated / redundant information, incomplete information, differential lag in reporting the cases, missing initial cases, the inclusion of imported cases as native cases, and partisan information. These all probes may lead to overestimation or underestimation of reproductive numbers. However, as the cases in this particular instance (www.covid19india.org) are chiefly pulled from official government handles, the extent of discrepancy may remain the same in some probes irrespective of the nature of the data source. Secondly, the investigators also tried to minimize these discrepancies by rigorous data cleaning, removal of imported cases (as reported) as far as possible by triangulating with other sources and subsequent merging of the final dataset. The estimates might be influenced by certain effect modifiers and confounders like population density, climatic variations and violation of assumption of random mixing. Conceptually, this phenomenon is dynamic and non-linear in nature and hence should be read with caution.

### Point 3:

In general, the work does not seem unreasonable, but the methods descriptions are generally lacking and need to be improved and expanded. It's also not clear how impactful the findings as presented are - lock-downs have been widely studied in COVID-19, and the general consensus already shows that they've decreased transmission. More contextualization to the situation in Indian and specific implications of this work would improve the manuscript.

**Response:**

**We thank the Reviewer for his/her encouraging words and suggestions for improving our manuscript. We have redrafted the Discussion section adding more context into the situation in India and the implications of our work. The relevant parts of the Discussion are provided below:**

The overall picture suggests the initial success of Indian states to curtail the rise of curve. This reduction can mainly be explained by reduced number of contacts among people owing to movement restrictions. Studies on the impact of lockdown in other countries also reported reduction in reproductive number which translates in to flattening of the curve and delaying of peak.[20-24] Yet as mentioned earlier, the time-varying reproduction number (R(t)) estimations are dynamic and may change over age structure, time and nature of intervention. Continuing nationwide lockdown will not be feasible in long term and restrictions have to be eased in phase wise manner. Therefore, in the post-lockdown era, it might be a challenge to maintain this path and this may be the period where the absolute burden of the infected persons will be high.[25, 26] Also, there has been disproportionately higher burden of serious infections including those requiring intensive-care among individuals more than 60 years of age as compared to younger adults.[27] This coupled with the higher prevalence of comorbid conditions (50%) in individuals over 60 years in India may warrant a strategy tailored to this section of population. [28] This also suggests that in addition to identification of infection, it is imperative to shift the focus on mortality prevention. Containment strategies like lockdown has given us the much-needed opportunity to delay the peak flatten the epidemic-curve. The time bought should be utilized to intensify the surveillance among `at-risk' individuals and buttress the health infrastructure including hospital beds with oxygen availability and critical care beds with ventilators and tele-medicine. [29-31]

# Reviewer #2:

The authors used an R package “R0” to estimate the reproduction number of COVID-19 in 10 states in India, 15 days and 30 days into the “lockdown” respectively, through a maximum likelihood method based on chain binomial models.

The authors used an R package “projections” to simulate 1000 probable epidemic outbreak trajectories, based on probability mass function dependent branching process assuming it follows a Poisson distribution. Based on data as of April 23, 2020, the authors projected the cumulative incidence 10 days into the future (May 3, 2020).

The authors demonstrate their command of advanced analytical skills through their analysis of the epidemiological data. However, there is room for improvement with regard to careful and clear explanations of concepts and codes therein.

**Response:**

**The authors would like to thank the Reviewer for his/her comments and suggestions in making our manuscript better. We tried to address all the issues raised and hope our responses are adequate.**

## Point 1:

Page 3: “The serial interval for COVID-19 … was used as time stamp for the estimation.” Could you please explain what do you mean by “time stamp” here?

Can you please clarify if you are using serial interval as a proxy for generation time?

**Response:**

**By “timestamp” we meant “time-step”. The authors apologize for the error in the text. We have used the serial interval as a proxy for generation time. We thank the Reviewer for pointing it out. We have rewritten the relevant part of the methodology as below:**

**Serial Interval**

For the parameter distribution function (PDF), we could not obtain the generation time (time lag between the infection in primary case and secondary cases) distribution directly with infector-infectee pairs due to the lack of data availability. Therefore, it was substituted with the serial interval distribution discretized on a 1-day time step. This was created using the generation.time()” function in the “R0” package. For parametrisation purposes we chose a gamma distribution as it accommodates for the underlying changing number of events in the constant event rate (Poisson process). The distribution assumptions were aligned with the emerging literature as well as the observed plausible transmission dynamics. The mean and standard deviation for serial interval approximations were 4.4 days and 3 days respectively.[14] The shape (number of events in time step) and scale (the reciprocal of event rate) of the distribution were 2.15 and 2.04 respectively.

## Point 2:

Page 3: Title of Table 1. Please include: “as of 23rd April 2020” in the title of Table 1. Because a table needs to be standalone and without the date of data it will not be meaningful.

**Response:**

**The authors apologize for the typographical error and rectified it in the resubmitted Manuscript.**

## Point 3:

Page 3: “We used the package incidence to model the incidence and estimate growth rate and doubling time and package R0 to estimate the reproduction number (R0) for different states. (7–9) The package projections was used to simulate the epidemic outbreaks and project their respective trajectories based on the state specific transmission parameters.(10)” Here the names of the R package, “incidence”, “R0” and “projections” should be in quotes. Otherwise, it is confusing to the readers.

**Response:**

**The authors apologize for the typographical error and rectified it in the resubmitted Manuscript.**

## Point 4:

Page 4: “v-line”: change to “vertical lines”

**Response:**

**The authors apologize for the typographical error and rectified it in the resubmitted Manuscript.**

## Point 5:

Page 4: Table 2: Is the range in square brackets, a range or a confidence intervals. Reproduction number for Tamil Nadu: why was it NA but with the range in square brackets?

**Response:**

**Table has been rectified now.**

## Point 6:

Page 5: Figure 1. Please provide more details in the legend of the figure. A figure should be standalone. The legend should explain that line graphs represent cumulative cases and bar charts represent new cases per day.

**Response:**

**The authors for the typographical error and rectified it in the resubmitted Manuscript. We have added the legend separately for both daily incidence and cumulative incidence. The revised Figure is given below:**

## Point 7:

Page 5: “baseline R0 (calculated at 15 days into lockdown) and effective R0 at 30 days into lockdown”: R0 is the symbol of the basic reproduction number, which is defined as the number of secondary cases caused by an index case in a totally susceptible population, without interventions or behavioral change. When India was in 15 days into lockdown or 30 days into lockdown, these assumptions no longer held. Therefore, the reproduction number 15 or 30 days into lockdown should be referred to as an effective reproduction number (Re), or a time-varying or time-dependent reproduction number (Rt). They are not R0 in these cases. Calling them “baseline R0” or “effective R0” are wrong. The comment here also applies to “effective R0” in the Discussion (e.g., Page 7, the 3rd line in the 1st paragraph in the Discussion; also in other places).

**Response:**

**Now we have used term R_(t)_ (time-varying reproduction number) for 15 and 30 days into lockdown. Changes have been made throughout the manuscript.**

## Point 8:

Page 7: R_t as the symbol for effective reproduction numbers is introduced. So why would you not use it consistently across the entire paper?

**Response: Now, we have used term R_(t)_ for effective reproduction number throughout the manuscript.**

## Point 9:

Page 7-8: The end of page 7: “to delay the peak flatten the epidemic-curve”: Please change to “to delay the peak and flatten the epidemic curve”.

**Response:**

**The authors for the typographical error and rectified it in the resubmitted Manuscript.**

## Point 10:

The following 2 medRxiv preprint manuscripts will be helpful to you in clarifying different concepts and the statistical methods that estimated them.

Practical considerations for measuring the effective reproductive number, Rt

doi: https://doi.org/10.1101/2020.06.18.20134858

A comparative analysis of statistical methods to estimate the reproduction number in emerging epidemics with implications for the current COVID-19 pandemic

doi: <https://doi.org/10.1101/2020.05.13.20101121>

**Response:**

**We are greatful to reviwers for suggesting these papers. These helped in revising the manuscript particularly with reference explanation of methods for estimation of reproduction numbers. We have cited these at appropriate sections in the manuscript.**

## Point 11:

Finally, the authors may want to reconsider the flow of their Discussion section. I would recommend the following flow:

Discussion paragraph #1: Highlights of the key results of the paper

Discussion paragraph #2: Situating your manuscript in the body of recent literature of COVID-19 epidemiology, esp. other papers that estimate the R_t of COVID-19

Discussion paragraph #3: Limitations of the paper

Discussion paragraph #4: Conclusions

**Response:**

**The authors thank the Reviewer for his/her suggestions. We have taken your advice and redrafted the Discussion section. We included recent papers on Indian context estimating time-varying reproduction number (R_(t)_). We also addressed the issue of crowd-sourced data in the limitations section of the Discussion. We hope the corrections made are adequate and address the issues raised.**

## Point 12:

Supplementary Appendix 1

Some of the codes go beyond the margin and there are incomplete in the PDF file (these happen multiple times in multiple pages). Please amend.

Section heading “Creating incidence objectes…”: “objectes” should be “objects”.

Section “Serial Interval”: “The serial interval is ceated using…”: “ceated” should be “created”.

Can we say that you are using the serial interval to approximate the generation time?

**Response:**

**We thank the Reviewer for his/her comments. We apologize for the typographical errors. We ensured that all the code stays within the margins of the PDF document. Regarding the Serial Interval being used as an approximation of Generation Time, we have mentioned the same in the Methodology section as suggested by the Reviewer. We have made the necessary corrections in the resubmitted manuscript and Supplementary Appendix.**

## Point 13:

Page 6:

\# Aggregate the timepoints after enforcment of lockdown

plot_states_14 <- readRDS("transmission_params_14.rds")

plot_states_30 <- readRDS("transmission_params_30.rds")

Where does the “transmission_params_14.rds” and “transmission_params_30.rds” files come from? I cannot find it in the previous code chunks.

Please make sure that you introduce them in the text and explain what they are.

**Response:**

**The “transmission_params_14.rds” and “transmission_params_30.rds” are summary tables of transmission parameters of 15 days and 30 days into the epidemic. These objects were created in the R code provided in Supplementary Appendix 1. We apologize for not considering the issue raised by the Reviewer earlier. We realise our mistake and amended it. We have provided the concerned *.rds files, their description and the corresponding code at the data repository at protocols.io where we provided the description and computational workflow along with the necessary files (**<https://protocols.io/view/incidence-modelling-covid19-computational-workflow-bh8vj9w6>)**. We also refined the R code chunks and explained them in greater detail in the updated Supplementary Appendix.**

## Point 14:

Supplementary Appendix 2

“Under Loading Packages & .rds files” What are the RDS files here? You need to introduce to your readers what they are: “df_backup.rds”, “transmission_params_30.rds”, and “states_keep.rds”.

To comply with PLOS ONE requirement to make the data underlying the findings in the manuscript fully available, have you considered also provide these RDS files as supplementary materials? Otherwise, even with your R codes, your analysis cannot be repeated by the readers.

**Response:**

**The authors apologize for overlooking the issue raised. We have taken your suggestions and elaborated on the RDS files and the corresponding R code. We have also submitted all relevant files necessary for reproducibility at protocols.io (**<https://protocols.io/view/incidence-modelling-covid19-computational-workflow-bh8vj9w6>)

**We have added the following text in the Supplementary Appendix 2:**

The df_new_05May.rds file contains line listing data till 5th May 2020. The variables are same as described in Supplementary Appendix 1. transmission_params_30.rds file contains the transmission parameters (time-varying reproduction number, doubling time, and growth rate) of the ten selected Indian states estimated after 30 days of lockdown. The states_keep.rds file contains the list of selected Indian states as mentioned in the manuscript and Supplementary Appendix 1.
